# Persistent low-level viremia and symptomatic HIV cerebrospinal fluid escape in a person living with HIV receiving darunavir: a case report

**DOI:** 10.1016/j.bjid.2026.105970

**Published:** 2026-09-26

**Authors:** Enrico Stefano Suriano, Karin Kuslevicius, Mariana Martins dos Santos Leal, Luiz Fernando Degrecci Relvas, Gustavo Arthur Reis Schneider

**Affiliations:** a Hospital Santa Marcelina, São Paulo, SP, Brazil; b Universidade de São Paulo (USP), São Paulo, SP, Brazil

**Keywords:** HIV, Central nervous system infections, Viral escape, Persistent low-level viremia

## Abstract

HIV Cerebrospinal Fluid (CSF) viral escape is a condition characterized by inadequate virological control within the CSF despite systemic antiretroviral therapy, often assessed through discordant CSF and plasma HIV viral loads. The clinical relevance of CSF viral escape remains poorly understood, but it is thought to contribute to a spectrum of neurological and neurocognitive manifestations. The present study details the case of a 51-year-old individual from São Paulo, Brazil, presenting with acute meningoencephalitis and seizures in the context of persistent low-level viremia. Differential diagnoses were considered to rule out other conditions, such as infectious and immune-related diseases, eventually leading to a diagnosis of HIV CSF viral escape, confirmed through clinical presentation and a series of diagnostic evaluations. Antiretroviral therapy was subsequently optimized to improve central nervous system penetration, resulting in clinical improvement, resolution of neurological symptoms and undetectable CSF viral load.

## Introduction

Central Nervous System (CNS) invasion by HIV has been described since the beginning of the HIV/AIDS epidemic. The mechanisms proposed to explain HIV neurotropism include the Trojan horse theory (virus-infected monocytes and T lymphocytes cross the blood-brain barrier), direct transcytosis (the virus infects epithelial cells and crosses the barrier) and disruption of epithelial tight junctions by the viral Tat protein.^1^ The consequences of CNS invasion are diverse, ranging from HIV-Associated Neurocognitive Disorder (HAND) to HIV Cerebrospinal Fluid (CSF) escape. HIV CSF viral escape, whose definition is heterogeneous in the literature, corresponds to a failure of virological control within the host’s CNS, assessed by quantifying HIV viral load in the CSF in comparison with the viral load in the plasma compartment. Among the hypotheses proposed to explain viral escape, the establishment and persistence of a viral reservoir in the CNS from early stages of HIV infection stands out; this reservoir remains latent, with the potential for eventual reactivation and viral replication.^2^

The main risk factors for the development of HIV CSF escape include poor adherence to Antiretroviral Therapy (ART), the use of antiretroviral agents with limited CNS penetration, the presence of antiretroviral resistance–associated mutations and a low CD4+ T-lymphocyte (CD4+ T-cells) nadir, particularly below 200 cells/µL.^3^ These factors may favor the selection of resistant viral variants and impair adequate plasma virological control, in addition to promoting HIV compartmentalization within the CNS. Furthermore, persistent low-level viremia^4^ (defined as plasma viral loads above the limit of detection and below 200 copies/mL on two or more consecutive measurements, representing an intermediate state between viral blips and virological failure) has been hypothesized as a risk factor for HIV CSF escape,^5^ as it may result from inadequate virological control and, thus, serve as an indirect marker of residual viral replication within sanctuary compartments.

HIV CSF escape has been receiving increasing clinical attention. However, its full impact in the CNS is not yet fully understood. We describe the case of a person living with HIV (PLWHIV) with persistent low-level viremia who developed primary symptomatic HIV CSF escape.

## Case report

The patient is a 51-year-old brown cisgender heterosexual man residing in the city of São Paulo (SP), Brazil. He was diagnosed with HIV infection approximately twenty years prior to hospital admission and had since maintained regular use of ART. His most recent ART regimen consisted of Darunavir/Ritonavir 800/100 mg once-daily (DRV/r 800/100) and lamivudine (3TC) 300 mg / Tenofovir (TDF) 300 mg once-daily. In the past, the patient had been treated with several antiretroviral agents, including Zidovudine (AZT), Nevirapine (NVP) and raltegravir (RAL). The most recent CD4+ T lymphocyte count was 540 cells/µL and the patient had maintained persistent low-level viremia throughout the years of treatment, with values consistently below 200 copies/mL (Fig. 1). The last HIV viral load prior to hospital admission was 56 copies/mL. Approximately twelve years before admission, HIV genotypic resistance testing was performed and showed no resistance-associated mutations.

**Fig. 1:**
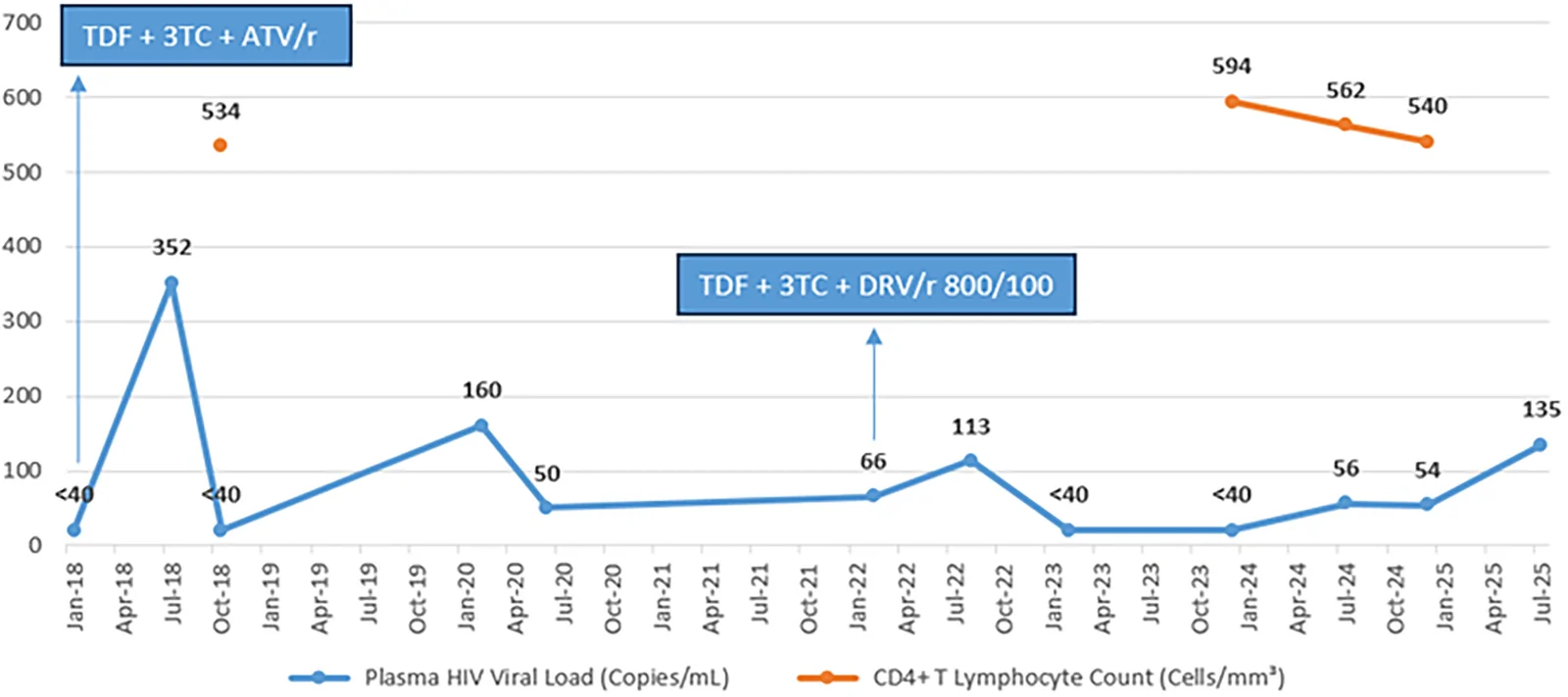
Persistent low-level HIV viremia and variation in CD4+ T-lymphocyte count during antiretroviral therapy. Note: 3TC, Lamivudine; ATV/r, Atazanavir boosted with ritonavir; DRV/r 800/100, Darunavir 800 mg boosted with Ritonavir 100 mg once-daily; TDF, Tenofovir.

His medical history was notable for tobacco use (100 pack-years) and alcohol use disorder, as well as prior use of cocaine (both intranasal and crack). He had discontinued the use of these substances three years prior to hospital admission. The patient also had structural abnormalities of the pulmonary parenchyma on chest computed tomography, suggestive of chronic obstructive pulmonary disease. In addition, his medical history included a bus-related pedestrian accident three years prior to hospitalization, resulting in traumatic brain injury and a right femoral fracture. There were no focal neurological deficits, and the fracture was managed conservatively; however, residual weakness and gait instability led to partial dependence in basic and instrumental activities of daily living. Finally, he had been hospitalized twenty days prior to the current admission during the investigation of chest pain without a positive troponin trend or obstructive lesions on coronary angiography, being discharged with a diagnosis of Troponin-positive Nonobstructive Coronary Arteries (TINOCA).

On an extensive epidemiological assessment, the patient reported an episode of upper respiratory tract infection 30-days prior to the current hospitalization, characterized by productive cough and dyspnea. He was evaluated at an external healthcare facility and managed with a prescription of amoxicillin, which led to resolution of symptoms. There was no history of travel in the preceding 12-months, contact with wild or domestic animals, any form of home cultivation, exposure to forested areas or streams/waterfalls/lakes, presence of standing water or accumulated waste in his neighborhood or any history of flooding in the region. The patient had no available records regarding his vaccination status. He resided near vacant lots and had access to adequate sewage and waste collection services in his neighborhood.

The patient was admitted to the emergency department of a tertiary general hospital due to an episode (as reported by family members) of abnormal eye movements and disorganized contractions of all four limbs, lasting a few minutes and followed by decreased level of consciousness, suggesting a seizure with a postictal period. He did not exhibit nuchal rigidity, fever or additional seizure episodes; however, he reported a single episode of frontal headache, managed at home with simple analgesics, without recurrence. On admission, his blood pressure was 125/93 mmHg, heart rate was 125 beats per minute, axillary temperature was 37.3°C and capillary blood glucose was 95 mg/dL. He remained with a reduced level of consciousness, with spontaneous eye opening, no interaction with the examiner, no verbal output and localization of painful stimuli (corresponding to a Glasgow Coma Scale score of 10). No focal neurological deficits or meningeal signs were detected. Physical examination of the remaining systems was unremarkable.

Admission laboratory tests revealed the following abnormalities: ionized calcium of 1.32 mmoL/L and C-reactive protein of 11.4 mg/dL. Hemoglobin, leukocyte/platelet, plasma glucose, venous blood gas analysis, creatinine, urea, sodium, potassium, magnesium and troponin levels were within normal limits. Serological testing was negative for hepatitis-B (non-reactive hepatitis-B surface-antigen, hepatitis-B surface-antibody and hepatitis-B core-antibody), hepatitis-C, syphilis and human T-Lymphotropic Virus (HTLV). Additional serologies showed negative IgM and positive IgG for Epstein-Barr virus, cytomegalovirus, toxoplasmosis and hepatitis A. The CD4+ T-cell count was 274 cells/µL.

Chest radiography showed no findings suggestive of an infectious process and the patient had no respiratory symptoms. Computed tomography of the abdomen and pelvis revealed no relevant abnormalities. Cranial computed tomography demonstrated moderate dilation of the supratentorial ventricular system, Sylvian fissures and temporal horns of the lateral ventricles (findings compatible with cerebrospinal fluid flow disturbance), as well as marked diffuse cortical atrophy, suggestive of ex-vacuum hydrocephalus. Given the hypothesis of structural epilepsy, carbamazepine 200 mg every 8-hours was initiated.

Brain magnetic resonance imaging revealed periventricular enhancement (bilateral frontal subcortical, right parieto-occipital and in the posterior horn of the right lateral ventricle, with cortical sparing), as well as pachymeningeal and pontine enhancement. Additionally, bilateral cerebellar hypointense foci were observed, reflecting cerebellar atrophy, along with reduced cerebellar volume relative to the remaining brain parenchyma (with prominence of the cerebellar folia). These findings are consistent with central nervous system atrophy and periventricular leukoencephalopathy. Fig. 2 illustrates the main abnormalities identified.

**Fig. 2:**
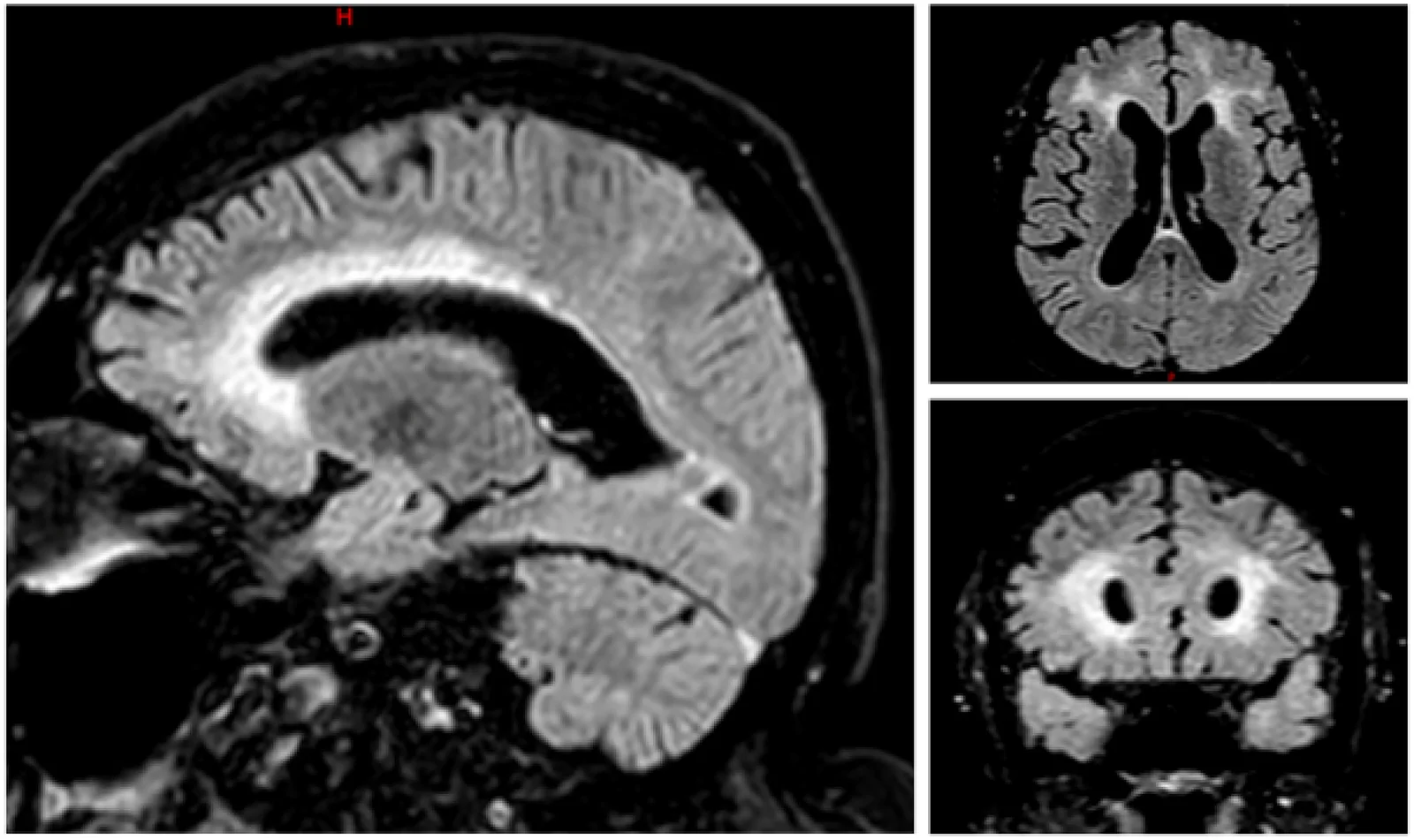
Brain magnetic resonance imaging demonstrating periventricular and pachymeningeal enhancement, suggestive of periventricular leukoencephalopathy.

A CSF analysis performed on hospital day-12 revealed a clear and colorless appearance, an opening pressure of 22 cmH_2_O, a leukocyte count of 16.3 cells/mm^3^ (with 88% lymphocytes on differential count), red blood cells of 1.3 mm^3^, protein concentration of 182.6 mg/dL and glucose level of 44 mg/dL (less than two-thirds of the capillary blood glucose, which was 96 mg/dL). Adenosine deaminase was 5.29 U/L. Negative results were obtained for Gram stain, Acid-Fast Bacilli (AFB) smear, *Mycobacterium tuberculosis* PCR, cultures (including fungi, aerobic/microaerophilic bacteria and mycobacteria), India ink staining (for *Cryptococcus* spp.) and VDRL. Urine Lateral Flow Lipoarabinomannan Antigen test (LF-LAM) was collected and yielded a negative result. Awake Electroencephalography (EEG) demonstrated continuous, diffusely and symmetrically disorganized background activity, without epileptiform discharges or seizures.

Based on clinical history and CSF findings, empiric therapy was initiated with intravenous acyclovir 10 mg/kg every 8-hours, intravenous ampicillin 2g every 4-hours, tuberculosis treatment (Rifampicin, Isoniazid, Pyrazinamide and Ethambutol ‒ RHZE) and weight-adjusted dexamethasone, targeting the diagnostic hypotheses of herpetic encephalitis, *Listeria monocytogenes* meningitis and tuberculous meningitis, respectively. Initiation of the RHZE regimen required adjustment of antiretroviral therapy due to the drug-drug interaction between darunavir and rifampicin. Accordingly, darunavir/ritonavir 800/100 mg once-daily was replaced with dolutegravir 50 mg twice-daily. In light of this ART modification and the interaction between the new regimen and carbamazepine (an inducer of cytochrome P450), topiramate 25 mg twice-daily was initiated, with the aim of gradually tapering carbamazepine until its discontinuation.

The possibility of arbovirus-related meningoencephalitis was considered; therefore, PCR and IgM testing for dengue, chikungunya and Zika viruses in the CSF were requested and yielded negative results. A repeat CSF analysis was performed, revealing a clear and colorless appearance, a leukocyte count of 8.6 cells/mm^3^ (with 74% neutrophils and 19% lymphocytes), red blood cells of 71.6 mm^3^, protein concentration of 109.8 mg/dL, glucose level of 54 mg/dL (less than two-thirds of the capillary blood glucose of 101 mg/dL), lactate of 1.7 mmol/L and adenosine deaminase of 1.49 U/L. Negative results were obtained for bacterioscopy, AFB smear, India ink staining, cryptococcal latex agglutination, cultures (including fungi, aerobic/microaerophilic bacteria and mycobacteria), VDRL and *Mycobacterium tuberculosis* PCR. Additional tests, including PCR for herpes simplex virus types 1 and 2, varicella-zoster virus, *Neisseria meningitidis, Streptococcus pneumoniae* and *Haemophilus influenzae*, were also negative. Furthermore, serum testing for antibodies against *Listeria monocytogenes* by tube agglutination was negative (titer <1:320). Given the prior history of persistent low-level viremia and the neuroimaging findings, paired analysis of HIV viral load in CSF and plasma was requested. The plasma HIV viral load was 89 copies/mL, whereas the CSF HIV viral load was 284 copies/mL.

After extensive laboratory investigation, all etiological studies for typical bacterial meningitis, herpes simplex virus types 1 and 2, varicella-zoster virus, *Mycobacterium tuberculosis* and fungal infections were negative (additional testing for other self-limited viral meningoencephalitides, such as enterovirus and adenovirus panels, were not performed due to resource limitations). In light of the diagnosis of HIV CSF escape and the absence of alternative etiologies, the RHZE regimen, ampicillin and acyclovir were discontinued, favoring the diagnostic hypothesis of primary HIV escape within the CNS. Discontinuation of rifampicin allowed for antiretroviral therapy adjustment, with regimen intensification to enhance CNS drug penetration: addition of zidovudine/lamivudine 300/150 mg twice-daily, continuation of dolutegravir 50 mg twice-daily, discontinuation of tenofovir 300 mg once-daily and reintroduction of darunavir/ritonavir 800/100 mg once-daily.

Finally, on hospital day-18, given the patient’s progressive clinical and neurological improvement, he was discharged with an adjusted ART regimen. Two months after discharge, the patient was readmitted due to infectious exacerbation of chronic obstructive pulmonary disease, without any neurological symptoms since the prior discharge. Thus, sustained improvement of the neurological condition was observed. A repeat plasma HIV viral load obtained during this readmission was undetectable. Six months after discharge, both plasma and CSF viral loads were undetectable, and the patient remained without any neurological symptoms.

## Discussion

We report the case of a PLWHIV with a history of persistent low-level viremia, receiving ART with a high-potency protease inhibitor (darunavir/ritonavir), who was hospitalized for acute meningoencephalitis and seizure breakthrough, underwent an extensive investigation of differential diagnoses and was ultimately diagnosed with symptomatic HIV CSF escape. Intensification of ART resulted in undetectable plasma and CSF viral loads and resolution of neurological symptoms. This case report provides important contributions to clinical practice, as it addresses a clinical entity that remains underrecognized, requires targeted diagnostic investigation and often demands tailored therapeutic management.

Although the first formal description of HIV CSF escape was published in 2003^6^ and its estimated prevalence is clinically relevant (with approximately 5–10% of PLWHIV experiencing episodes of HIV CSF escape),^7^ evidence regarding its true prevalence and impact in Brazilian and Latin American populations remains limited. A study by Christo et al.^8^ allows for a rough estimation of prevalence in Brazil: among 90 PLWHIV who underwent lumbar puncture, HIV RNA was detected in the CSF in 50 (55.6%), with higher rates observed in patients with opportunistic neurological diseases compared with those without neurological conditions (63.4% vs. 26.3%, respectively). However, paired CSF and plasma analyses were not performed, precluding accurate assessment of the number of viral escape cases within that cohort. In addition to the substantial scarcity of Latin American epidemiological data, heterogeneity in access to CSF HIV viral load testing across healthcare settings further hampers confirmatory diagnosis of HIV CSF escape in many regions.

The case presented in this report exhibited a hyperacute evolution of symptoms, with a short interval between the onset of clinical manifestations and the search for medical care. Although an acute course is classically described, most cases present with a subacute course, evolving over days to weeks. Therefore, the possibility of distinct patterns of clinical presentation should be considered in the recognition of this condition.^3^^,^^9^ Regarding radiological findings, the pattern observed was that of bilateral and symmetric leukoencephalopathy, which corresponds to one of the most typical phenotypes of this condition,^10^ reflecting a diffuse process of inflammation and white matter dysfunction. Thus, although nonspecific, the detection of such a pattern in PLWHIV should raise suspicion for HIV CSF escape and prompt a targeted diagnostic approach. Finally, the CSF findings in this case (pleocytosis, elevated protein levels, hypoglycorrhachia and increased opening pressure) were nonspecific, reflecting an active meningoencephalitic inflammatory state that is potentially observed in other CNS infections, such as tuberculous meningitis and *Listeria monocytogenes* meningitis. In the Brazilian context, where opportunistic infections and neuroinfections remain significant causes of hospitalization among PLWHIV,^11^ it is essential that HIV CSF escape be considered a relevant differential diagnosis in this population presenting with acute neurological manifestations and an inflammatory CSF profile, in order to avoid diagnostic and therapeutic delays.

Persistent low-level viremia may be associated with HIV CSF escape, indicating ongoing viral replication in compartments where ART efficacy is limited, thereby characterizing incomplete virological suppression. Within the CNS, this phenomenon may result from viral replication in resident cells such as macrophages and microglia, as well as from the limitations imposed by the blood-brain barrier on antiretroviral penetration and distribution, favoring the emergence of resistant viral variants and the occurrence of viral escape.^12^ Accordingly, persistent low-level viremia may reflect intermittent viral release from these reservoirs or the selection of compartment-adapted variants with characteristics distinct from those circulating in plasma.^13^ This process is associated with sustained intrathecal immune activation and local production of inflammatory mediators, creating an environment that leads to neuronal dysfunction and the development of neurological symptoms. Therefore, we support the hypothesis that persistent low-level viremia should be interpreted as a risk factor for HIV CSF escape, reinforcing the importance of selecting ART regimens with adequate penetration into the cerebral compartment.

With regard to ART formulations, there is evidence that certain antiretroviral agents may be more strongly associated with HIV CSF escape. According to Mukerji et al.,^14^ treatment with less contemporary protease inhibitors (such as atazanavir and unboosted protease inhibitors) in combination with reverse transcriptase inhibitors is an independent predictor of HIV CSF escape in treatment-experienced patients. Currently, with the use of integrase strand transfer inhibitors as first-line ART regimens, as well as the use of potent protease inhibitors (such as ritonavir-boosted darunavir), the overall risk is likely lower than that previously reported in the literature. In addition to the choice of antiretroviral agent, drug dosing should also be considered, as although the safety and non-inferiority of darunavir/ritonavir at a dose of 800/100 mg once-daily for virological control in PLWHIV have been well established, this dosage may have implications for CNS penetration and the occurrence of viral escape. One study^15^ calculated the inhibitory quotient (the ratio between drug concentration in the CSF and the inhibitory concentration required to block 95% of HIV replication in vitro) of darunavir at different dosing regimens and found that the quotient for the 600 mg twice-daily dose (18.5) was higher than that for the 800 mg once-daily dose (8.2), suggesting greater CNS penetration with twice-daily administration. The present case might be an example of this scenario, as the patient did not achieve adequate plasma virological control, possibly favoring the occurrence of CSF viral escape due to suboptimal CSF penetration of darunavir at the administered dose (although we could not confirm this hypothesis due to unavailability of darunavir CSF and plasma concentration testing). To date, we have not identified reports in the literature describing HIV CSF escape in PLWHIV receiving darunavir at a dose of 800 mg once-daily or specifically addressing the role of darunavir dosing in the occurrence of viral escape. Nevertheless, darunavir maintains high CNS penetration and potency compared with other antiretroviral classes and other protease inhibitors and therefore remains an indispensable tool in the contemporary management of PLWHIV.

There was marked neurological improvement following ART intensification with zidovudine and dolutegravir, as expected for agents with greater CNS penetration. According to these management strategies in HIV CSF escape, the Neuro+3 study^16^ for ART management in PLWHIV with confirmed HAND demonstrated that ART intensification guided by an increase in the CNS Penetration Effectiveness (CPE) score by three or more points and a total CPE score of nine or higher led to improved neuropsychological performance and reduced cognitive impairment after 48 to 96-weeks of treatment. An observational study^17^ evaluated two management strategies for HIV CSF escape associated with neurological manifestations (ART intensification with the addition of zidovudine vs. switching to regimens with improved CNS penetration, involving a boosted protease inhibitor with or without an integrase inhibitor) and found that both approaches resulted in reduction or suppression of CSF viral load and neurological improvement in most patients (78% vs. 71%, with no clear superiority of one strategy over the other), suggesting that ART optimization directed toward the CNS compartment is effective in controlling neurosymptomatic viral escape. Finally, the possibility of performing repeat genotypic resistance testing in this patient to identify resistance-associated mutations was considered; however, the low viral load precluded this approach.

This clinical case involved a patient with a plasma HIV viral load of 89 copies/mL (1.9494 log₁₀) and a CSF viral load of 284 copies/mL (2.4533 log₁₀), resulting in a CSF viral load exceeding the plasma viral load by 0.5039 log₁₀. According to several authors, there is no consensus in the medical literature regarding the diagnostic criteria for HIV CSF escape. One study comparing different diagnostic criteria^18^ demonstrated significant heterogeneity in the number of diagnoses made within the same sample, depending on the criterion applied, ranging from 9% to 38.9%. These criteria range from simple plasma-CSF discordance (detectable CSF levels with undetectable plasma levels) to specific quantitative thresholds, such as CSF RNA levels exceeding plasma levels by 0.5 log₁₀ or by 1 log₁₀. Thus, although the patient described presented with prominent neurological symptoms and significant radiological abnormalities, some of the currently proposed criteria would have failed to diagnose viral escape, while others would have been at the threshold of a false-negative result. Therefore, we favor diagnosing viral escape based on any detectable CSF HIV RNA when plasma HIV RNA is undetectable, or on CSF HIV RNA levels exceeding plasma levels to any degree when plasma HIV RNA is detectable, as proposed by Winston et al.,^19^ in order to reduce the number of false-negative results, which may lead to diagnostic delays and loss of therapeutic opportunity. It should be noted that CSF HIV RNA is an indirect measure of viral presence within the brain parenchyma; therefore, a low CSF viral load should not be interpreted as unequivocal evidence of viral absence in the CNS. Moreover, given that the natural history of HIV infection is characterized by plasma viral loads exceeding CSF viral loads,^20^ any reversal of this relationship should be considered pathological.

This report aimed to present a case of HIV CSF escape in a patient with persistent low-level viremia and to discuss the need for improved diagnostic tools (or broader access to existing ones) as well as increased awareness among healthcare professionals regarding viral escape. In addition, we argue in favor of broader and less restrictive diagnostic criteria to reduce the risk of false-negative results. The limitations of this work include the minimal sample size (which limits generalizability and increases susceptibility to undetected confounding factors), the exclusively descriptive nature of the study and the inability to perform repeat genotypic resistance testing to investigate viral resistance. The strengths of this report include the novelty of the case described, strict adherence to CARE guidelines, a detailed, comprehensive and chronological clinical description, the assessment of CSF viral load on follow-up, the exclusion of a wide range of differential diagnoses and the illustrative value of the case in questioning best practices related to the diagnosis and timely management of HIV CSF escape.

## Informed consent

The patient and his wife (witness) provided their informed consent and signed the Informed Consent Form.

## Funding

There was no need to purchase or acquire specific materials for the conduct of this study. Operational expenses, such as transportation and internet use, were covered by the researchers.

## Data availability statement

The data that support the findings of this study are available from the corresponding author upon reasonable request.

## Conflicts of interest

The authors declare no conflicts of interest.

## References

[1] Calado M., Ferreira R., Pires D., Santos-Costa Q., Anes E., Brites D. (2024). Unravelling the triad of neuroinvasion, neurodissemination, and neuroinflammation of human immunodeficiency virus type 1 in the central nervous system. Rev Med Virol.

[2] Said N., Venketaraman V. (2025). Neuroinflammation, blood-brain barrier, and HIV reservoirs in the CNS: an in-depth exploration of latency mechanisms and emerging therapeutic strategies. Viruses.

[3] Mastrangelo A., Turrini F., de Zan V., Caccia R., Gerevini S., Cinque P. (2019). Symptomatic cerebrospinal fluid escape. AIDS.

[4] The Ontario HIV Treatment Network. (2022). Rapid response service. Low-level HIV viremia: definitions, predictors, mechanisms, and clinical outcomes. https://www.ohtn.on.ca/wp-content/uploads/2022/01/RR166_Low-level-viremia_version2.pdf.

[5] Mukerji S.S., Misra V., Lorenz D.R., Cervantes-Arslanian A.M., Lyons J., Chalkias S. (2017). Temporal patterns and drug resistance in CSF viral escape among ART-experienced HIV-1-infected adults. J Acquir Immune Defic Syndr.

[6] Wendel K.A., McArthur J.C. (2003). Acute meningoencephalitis in chronic human immunodeficiency virus (HIV) infection: putative central nervous system escape of HIV replication. Clin Infect Dis.

[7] Guha D., Misra V., Yin J., Gabuzda D. (2023). CSF inflammation markers associated with asymptomatic viral escape in cerebrospinal fluid of HIV-positive individuals. Viruses.

[8] Christo P.P., Greco D.B., Aleixo A.W., Livramento J.A. (2007). Factors influencing cerebrospinal fluid and plasma HIV-1 RNA detection rate in patients with and without opportunistic neurological disease during the HAART era. BMC Infect Dis.

[9] Chan T.Y.H., de Zan V., Gregg A., Alagaratnam J., Gerevini S., Antinori A. (2021). The symptomatology of cerebrospinal fluid HIV RNA escape: a large case-series. AIDS.

[10] Narvid J., Callen A., Talbott J., Uzelac A., Dupont S.M., Chow F. (2018). Brain MRI features of CSF human immunodeficiency virus escape. J Neuroimaging.

[11] Telles J.P., Fernandes R., Barros T.D., Maestri A., Vitoriano T., Borges L. (2021). Neurological manifestations in people living with HIV/AIDS in the late cART era: a prospective observational study at a tertiary healthcare center in São Paulo, Brazil. HIV Res Clin Pr.

[12] Kincer L.P., Dravid A., Trunfio M., Calcagno A., Zhou S., Vercesi R. (2024). Neurosymptomatic HIV-1 CSF escape is associated with replication in CNS T cells and inflammation. J Clin Invest.

[13] Trunfio M., Joseph S.B., Ghisetti V., Romito A., Martini C., Cusato J. (2018). Symptomatic cerebrospinal fluid HIV-1 escape with no resistance-associated mutations following low-level plasma viremia. J Neurovirol.

[14] Mukerji S.S., Misra V., Lorenz D.R., Uno H., Morgello S., Franklin D. (2018). Impact of antiretroviral regimens on cerebrospinal fluid viral escape in a prospective multicohort study of antiretroviral therapy-experienced human immunodeficiency virus-1-infected adults in the United States. Clin Infect Dis.

[15] Calcagno A., Simiele M., Alberione M.C., Bracchi M., Marinaro L., Ecclesia S. (2015). Cerebrospinal fluid inhibitory quotients of antiretroviral drugs in HIV-infected patients are associated with compartmental viral control. Clin Infect Dis.

[16] Force G., Ghout I., Ropers J., Carcelain G., Marigot-Outtandy D., Hahn V. (2021). Improvement of HIV-associated neurocognitive disorders after antiretroviral therapy intensification: the Neuro+3 study. J Antimicrob Chemother.

[17] Dravid A.N., Gawali R., Betha T.P., Sharma A.K., Medisetty M., Natrajan K. (2020). Two treatment strategies for management of neurosymptomatic cerebrospinal fluid HIV escape in Pune, India. Medicine.

[18] Trunfio M., Pinnetti C., Focà E., Bai F., Maffongelli G., Celani L. (2019). Cerebrospinal fluid HIV-1 escape according to different thresholds and underlying comorbidities. AIDS.

[19] Winston A., Antinori A., Cinque P., Fox H.S., Gisslen M., Henrich T.J. (2019). Defining cerebrospinal fluid HIV RNA escape. AIDS.

[20] Ulfhammer G., Edén A., Antinori A., Brew B.J., Calcagno A., Cinque P. (2022). Cerebrospinal fluid viral load across the spectrum of untreated human immunodeficiency virus type 1 (HIV-1) infection: a cross-sectional multicenter study. Clin Infect Dis.

